# Femoral Neck Fractures Treated by Closed Reduction and Internal Fixation with the Double Fluoroscope Technique: A Preliminary Study

**DOI:** 10.3390/jcm13051418

**Published:** 2024-02-29

**Authors:** Hyun Hee Lee, Kyung-Yil Kang, Seung-Yong Sung, Soo-Bin Lee, Sang-Hee Kim, Su-Il Jung, Dong Hyuk Shin, Byung Hak Oh, Dong-Sik Chae

**Affiliations:** 1 Department of Orthopedic Surgery, Catholic Kwandong University College of Medicine, International St. Mary’s Hospital, Incheon 22711, Republic of Korea; emprany@ish.ac.kr (H.H.L.); fbdlxk@naver.com (K.-Y.K.);; 2 College of Medicine, Yonsei Graduate School, Seoul 03722, Republic of Korea; 3 College of Medicine, Catholic Kwandong Graduate School, Gangneung-si 25601, Republic of Korea; 4 Department of Orthopedic Surgery, Konyang University Hospital, Daejeon 35365, Republic of Korea

**Keywords:** femoral neck fractures, closed reduction, internal fixation, double C-arm, radiation exposure reduction

## Abstract

**Background:** Fractures of the femur require significant radiation exposure during operations using fluoroscopy (C-arm), posing a high risk of radiation exposure to the medical staff and patients. To address this concern, in this study, we investigated the efficacy of using two fluoroscopy machines simultaneously. **Methods:** We categorized 30 patients with femoral neck fracture (FNF) into single and double C-arm groups. The operation and radiation exposure times during a closed reduction and internal fixation operation were investigated to evaluate whether the operation and radiation exposure times were effectively audited when the operation was performed using a double C-arm. **Results:** The total operation times were 93.21 ± 20.70 min and 66.69 ± 13.97 min for the single and double C-arm groups, respectively. Additionally, the total radiation times were 100.43 ± 24.59 s and 83.06 ± 19.53 s for the single and double C-arm groups, respectively. Operation and radiation exposure times in the two groups showed statistically significant differences (*p* < 0.05). **Conclusion:** The use of double C-arm in FNF can reduce operation and radiation exposure times. Hence, using the double C-arm in surgical treatment could reduce the risk of radiation exposure to medical staff and patients.

## 1. Introduction

The global incidence of hip fractures is increasing with the aging population, along with the increasing prevalence of osteoporosis. Projections indicate an increase to 2.6 million by 2025 and 4.5 million by 2050 [1]. The high incidence rate was attributed to falls in older patients with osteoporosis; however, there has been a recent increase in the number of relatively young patients aged 40–50 years with femoral neck fractures (FNF) resulting from high-energy trauma.

Arthroplasty is the preferred treatment for FNF in older patients, whereas closed reduction and internal fixation (CRIF) is preferred in younger patients because of several advantages, including the preservation of their joints and ease of operation facilitated by fluoroscopy (C-arm) [2,3].

In orthopedic practice, such as in the treatment fractures, fluoroscopy is widely used to minimize surgical invasiveness and reduce operation time. Orthopedic surgeons have shown considerable concerns regarding the side effects of radiation exposure during orthopedic procedures [4,5,6,7,8,9]. However, many orthopedic surgeons lack sufficient information regarding radiation physics and its deleterious effects. Surgeons should be aware of the harmful effects of radiation and maintain a dose as low as reasonably achievable [4,5,6,7,8,9]. In particular, proximal femoral fracture surgery results in higher radiation exposure than that of other orthopedic surgeries. Additionally, orthopedic medical staff exposed to high radiation levels have an elevated risk of cancer [5,6,7].

To minimize this risk, studies have suggested a reduction in operation and radiation exposure times in kyphoplasty, intertrochanteric fracture (cephalomedullary nailing), and FNF (cannulated screw fixation) surgeries using a double C-arm fluoroscope. However, research supporting the effectiveness of using a double C-arm is still lacking. Therefore, This study aimed to evaluate the efficacy of reducing the preparative, intraoperative, and radiation exposure times by simultaneously using a double C-arm during surgical treatment of FNFs [4,6,8,9].

## 2. Materials and Methods

### 2.1. Participants

The Institutional Review Board of the Catholic Kwandong University International Saint Mary’s Hospital approved this study (No. IS17RIMI0017). The study involved 30 participants who underwent CRIF using metal screws for FNFs at the Catholic Kwandong University International Saint Mary’s Hospital between 2017 and 2018. Patients who underwent arthroplasty or open-reduction internal fixation surgery were excluded from this study.

### 2.2. Operation Device

In this study, FNF surgeries were performed using 6.5 or 7.3 mm metal cannulated screws (C&S Medical Co., Ltd., Pocheon-si, Republic of Korea, and AO Foundation, Davos, Switzerland). A Siemens ARCADIS Varic fluoroscope (München, Germany) was used as the radiation device.

### 2.3. Study Design

All the participants provided informed consent for the use of their personal information. Subsequently, the participants were randomly assigned to the following two groups: one group utilizing a single fluoroscopy device and the other group employing double fluoroscopy devices.

A skilled senior orthopedic surgeon performed all the surgeries. Another senior orthopedic surgeon (B.H.O) and medical staff (who did not participate in the operation) evaluated the collected data. The evaluator was unaware of the group to which the collected data belonged.

### 2.4. Procedure

**Figure 1:**
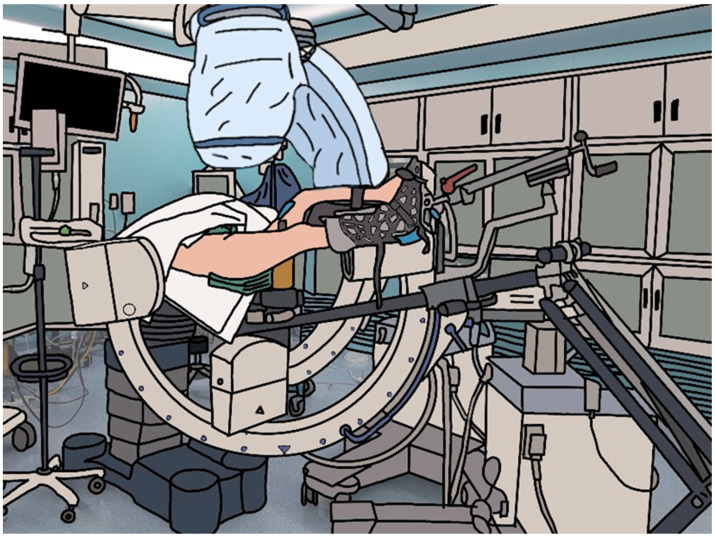
Preoperative patient and double C-arm positioning.

General or spinal anesthesia was administered preoperatively. The anesthetized patient was transferred to a fracture table, and the operating foot was secured to the fracture table boot. The non-surgical leg was fixed in a lithotomy position to facilitate smooth movement of the fluoroscopy device [6,9]. The arm on the surgery side was positioned laterally to enable the fluoroscopy device to capture a smooth axial image. In the group using double fluoroscopes, the first fluoroscope was placed between the legs of the patients and rotated to the axial plane with the hip joint center. Subsequently, the X-ray tube of the fluoroscope was placed near the injured hip with an approximate 20° tilt in the sagittal plane. The C-arm was rotated between 5° and 25° back toward the anteroposterior (AP) view from a flat position to ensure a true axial image of the femoral head and neck. A second fluoroscope was introduced from the uninjured leg and positioned vertically in the AP plane. The second fluoroscope was placed on the C-arm of the first fluoroscope. After performing an approximate closed reduction assisted by fluoroscopy, the degree of reduction at the FNF site was evaluated [6,9] (Figure 1). In the single fluoroscopic group, a single fluoroscope was positioned between the legs of the patients to obtain a true AP and axial view of the proximal femur by preoperatively relocating the fluoroscope back and forth. After performing a closed reduction assisted by fluoroscopy, the degree of reduction at the FNF site was evaluated [6,9,10,11,12,13].

**Figure 2:**
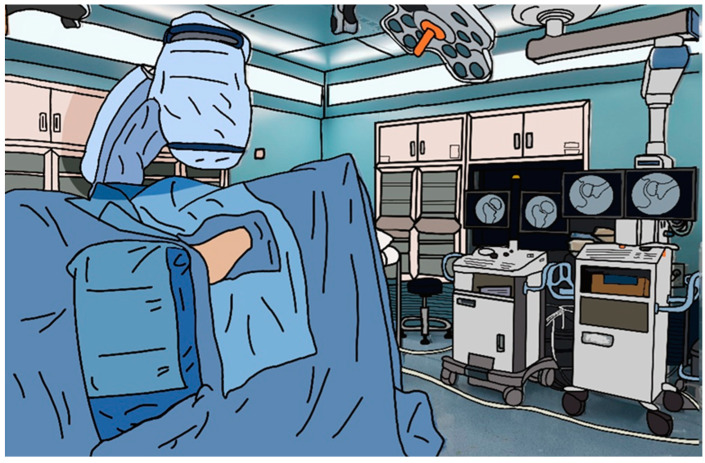
Positioning the patient and double C-arm during the operation.

After skin preparation, patients in the double fluoroscope group underwent sterile draping. In this process, the first fluoroscope was covered with surgical drapes, and the image intensifier of the second was covered with a separate sterile vinyl bag. During the surgery, AP and axial images could be obtained simultaneously without changing the position of the fluoroscope [6,9] (Figure 2). Patients in the single fluoroscope group underwent sterile draping, and the radiology technician repositioned the fluoroscope intraoperatively as required by the surgeon.

**Figure 3:**
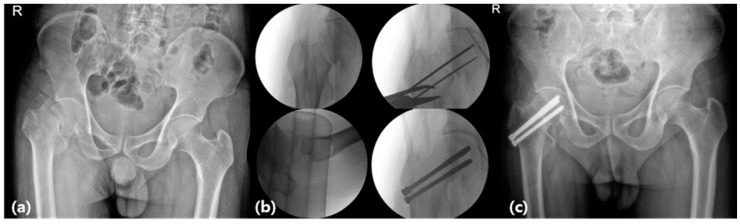
Operation for a femoral neck fracture. (**a**) Preoperative X-ray (anteroposterior view of both hips), (**b**) intraoperative fluoroscopy, and (**c**) closed reduction and internal fixation (cannulated screw). Abbreviation: R, right side

During FNF surgery, the reduction in the fracture site was confirmed through the C-arm by adjusting the degree of internal and external rotation of the fracture table. Subsequently, an incision was made approximately 3 cm below the trochanter, exposing the lateral cortical bone. Three Kirschner wires were inserted into the femoral bone head in an inverted triangle along the longitudinal axis of the femoral neck at 135° [6,9,10,11,12,13]. After insertion, the Kirschner wire was positioned 1 cm from the articular femoral head surface using a C-arm. We verified the position and length of the Kirschner wire using a C-arm and tightened the fracture by rotating three 6.5 or 7.3 mm-diameter cannulated screws. Finally, we checked for any abnormalities during passive movement of the hip joint, and the surgical site was cleaned and sutured to conclude the operation [10,11,12,13] (Figure 3).

### 2.5. Data Collection

We recorded the preparative time (from induction of anesthesia to incision), intraoperative time (from incision to wound closure), and radiation exposure times from the fluoroscope devices (by recording the usage time in seconds for every single push of the fluoroscope) at the end of the preparative and intraoperative phases. The radiation exposure time measured during the preparative period was the duration from anesthesia to incision and from C-arm installation to closed reduction at the fracture site. During surgery, the radiation exposure time was calculated as the radiation exposure duration from incision to suturing. Additionally, data on sex, age, body mass index, anesthesia method, and fracture type of the patients (Garden classification) were collected.

### 2.6. Statistical Analysis

All data were analyzed using IBM SPSS Statistics for Windows, version 22.0 (IBM Corp., Armonk, NY, USA). The single and double fluoroscopic groups were compared using the independent sample *t*-test or Mann–Whitney U test for continuous variables and the chi-square test for categorical variables. Statistical significance was set at values of *p* < 0.05.

## 3. Results

### 3.1. Characteristics of the Participants (Single C-Arm versus Double C-Arm)

This study involved 30 patients with FNF. The single C-arm group comprised 10 men and 4 women, and the double C-arm group comprised 8 men and women each. The average ages were 48.57 ± 20.01 and 50.94 ± 6.21 years for the single and double C-arm groups, respectively. The average body mass indexes were 22.22 ± 2.27 kg/m^2^ and 23.57 ± 3.07 kg/m^2^ for the single and double C-arm groups, respectively. Additionally, the anesthesia method and fracture classification of both the groups were investigated, revealing no significant differences between them (Table 1).

### 3.2. Comparison of Operation and Radiation Exposure Times between the Two Groups (Single and Double C-Arm Groups)

**Figure 4:**
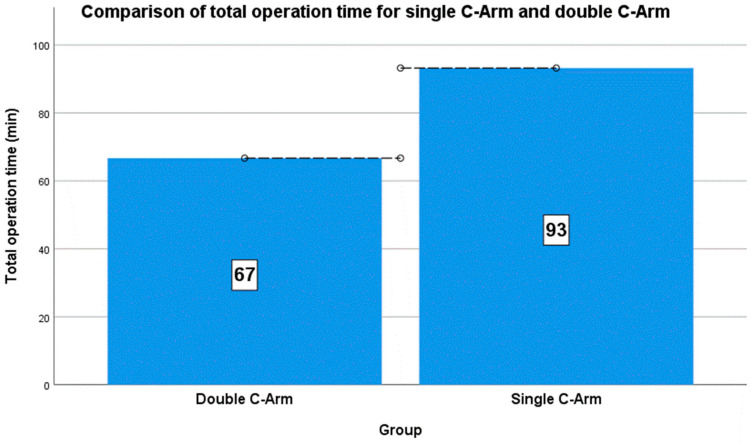
Comparison of the total operation time between the two groups.

The preparative time (min) for both groups was measured as the time required from after anesthesia to incision, and the operation time (min) was measured from incision to suturing. The average preparative times were 37.86 ± 6.99 min and 28.75 ± 11.47 min for the single and double C-arm groups, respectively, and the average operation times were 55.36 ± 17.26 min and 37.94 ± 6.55 min for the single and double C-arm groups, respectively. The total operation times were 93.21 ± 20.70 min and 66.69 ± 13.97 min for the single and double C-arm groups, respectively. The mean difference between both groups was approximately 26.52 min, with the single C-arm group requiring a longer operation time (Figure 4). A comparison of the means of both groups revealed significant differences of 0.001 (*p* <0.05) (Table 2).

The preparative radiation time (s) for each group was measured as the time required from after anesthesia to incision. The operation radiation time (s) was measured from incision to suturing. The average preparative radiation times were 19.93 ± 9.02 s and 14.25 ± 7.31 s for the single and double C-arm groups, respectively, and the average intraoperative radiation times were 80.50 ± 17.65 s and 68.00 ± 14.23 s for the single and double C-arm groups, respectively. The total operation radiation times were 100.43 ± 24.59 s and 83.06 ± 19.53 s for the single and double C-arm groups, respectively.

**Figure 5:**
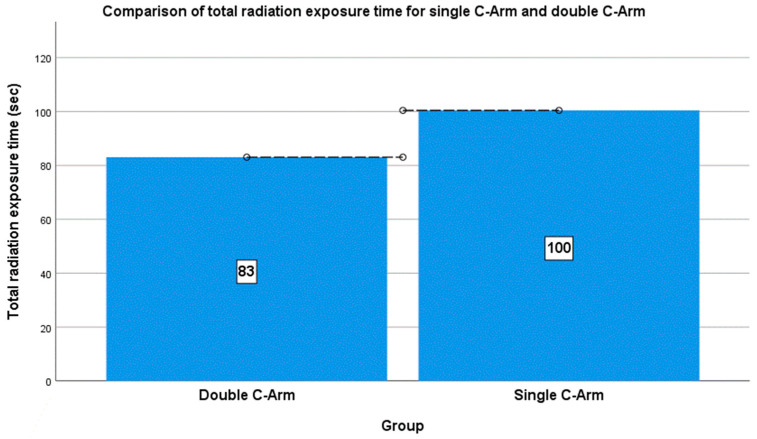
Comparison of total radiography exposure times between the two groups.

The mean difference between the two groups was approximately 17.37 s, with the single C-arm group requiring more operative radiation time than that of the double C-arm group (Figure 5). A comparison of the means of the two groups revealed a significant difference of 0.015 (*p* < 0.05) (Table 2).

## 4. Discussion

In this study, we investigated whether using a double C-arm reduces the operation and radiation exposure times during cannulated screw fixation surgery for FNFs. The total operation and radiation exposure times were 66.69 min and 83.06 s using the double C-arm and 93.21 min and 100.43 s using the single C-arm, respectively. Compared with the single C-arm, using the double C-arm reduced operation and radiation exposure times by approximately 26.52 min and 17.37 s, respectively. This study demonstrated that the double C-arm enables simultaneous viewing of AP and axial images, thereby facilitating reduced closed reduction, guide pin insertion, and cannulated screw fixation during femoral neck operations. Additionally, the need for additional radiation projection to obtain a proper image and the time consumed in moving the C-arm from the AP imaging position to the axial imaging position during surgery is eliminated.

Our results are consistent with those of previous studies. In a study by Gülenç et al. [9], performing surgery using a double C-arm for cannulated screw fixation for FNF resulted in an average reduction in operation and radiation times by 14.2 min and 24.5 min, respectively, as opposed to using a single C-arm, with a significant difference between the groups [9]. However, the measured radiation time in our study represented the total operating time of the equipment, and differences between the measured radiation exposure time (s) and the measurement method were observed whenever the imaging button of the radiation equipment was pressed [9]. Additionally, the preparative time measured from the reduction in the fracture site after anesthesia was 20.6 min and 18.3 min using double and single C-arms, respectively, with slightly more preparative time being required when the opposite double C-arm was used; however, no significant difference was observed [9]. A study by Brin et al. [6] focusing on femur intertrochanteric fractures using double and single C-arms during the operation demonstrated a significant difference between radiation exposure (average 14.6 s) and operation (average 10.4 min) times. The study suggested that using a double C-arm could reduce radiation exposure time, providing health benefits to patients and medical staff [6].

Fluoroscopy in orthopedic surgery units exposes patients and medical staff to a potentially detrimental amount of radiation. Patients are primarily affected by the radiation generated in the path between the X-ray tube and the image intensifier. However, medical staff are mainly affected by the scattered radiation generated by objects in the path between the X-ray tube and image intensifier when the primary radiation is emitted [14,15]. Sanders et al. [16] conducted a study to measure the amount of radiation exposure to orthopedic surgeons using a thermoluminescent dosimeter during fluoroscopy during intramedullary nailing, open reduction and internal fixation (plate and screw), and external fixation [16]. Their findings indicated that when fluoroscopy use exceeded 1.7 min, radiation exposure increased more rapidly than that at usage durations below 1.7 min. Additionally, they reported that protective equipment should be worn when using the radiation devices and suggested that exposure to radiation decreases when the distance from the radiation-emitting device increases [16]. In a similar study, Mahajan et al. [5] measured the amount of radiation exposure to different parts of the human body (the neck, chest, wrist, and gonads) for each orthopedic doctor based on the use of fluoroscopy during orthopedic surgery.

Based on the results of this study, the amount of radiation measured at the wrist exhibited positive correlation with the operation time [5]. Consequently, using a double C-arm to shorten the operation time is an effective method for reducing radiation exposure.

Several studies have reported that fluoroscopy can increase the likelihood of tumors in orthopedic surgeons and patients exposed to ionizing radiation [16,17,18,19]. Therefore, it is important to wear appropriate protective equipment and reduce radiation exposure time when using radiation devices [5,16,18,19]. A study examining tumor incidence accumulated over 24 years revealed that orthopedic surgeons have the highest risk of developing tumors [18]. Studies have shown that the amount of radiation exposure is higher in the spine and hips than in the limbs [20].

Many studies have explored the risk of ionizing radiation exposure as well as the reduction in surgical time and radiation exposure time, particularly in areas such as the spine and hips [6,9,21,22]. However, studies on the reduction in operation and radiation exposure times using a double C-arm in FNFs are limited. Several studies evaluating the effect of different implants used in FNFs, have demonstrated reduced operation time and bleeding with cannulated screw fixation or use of femoral neck system [13,23]. This result suggests that reducing operation time can reduce the amount of bleeding. Using a double C-arm in surgery for FNF is associated with reduced bleeding due to decreased operation time. However, the extent of bleeding was not investigated in this study. Nevertheless, positive results using a double C-arm were obtained when compared with similar studies [6,9,13].

In addition, there are studies that analyze the amount of radiation exposure and surgical accuracy by using the cone-beam computed tomography device for surgery of spine and pelvic acetabular fractures [24,25,26]. The cone-beam computed tomography device is smaller than the existing computed tomography device and can be used in the operating room due to its high utilization [24]. It was reported that the use of the cone-beam computed tomography device during surgery for acetabular fractures can reduce the gap and surgical accuracy of the bone fragment, and reduce the amount of radiation exposure compared to the existing computed tomography exam after open reduction internal fixation surgery [25]. These findings have shown the possibility of using cone-beam computed tomography for FNF, but related findings are lacking. Further studies on its use may be needed.

This study has some limitations. First, this study was conducted involving a small number of participants. Second, we did not measure ionizing radiation using a thermoluminescent dosimeter. Third, only the operation and radiation exposure times for single and double C-arm use were investigated. Fourth, the short and medium-term postoperative outcomes were not investigated. The persuasive power of our findings may be insufficient because of these limitations. Therefore, further studies with more participants are needed to confirm the advantages of surgery using double C-arm. Additional studies are also needed to measure the radiation exposure time and dose of ionizing radiation using a thermoluminescent dosimeter. Moreover, it is necessary to investigate the effects of the reduction in operation and radiation exposure times due to the use of double C-arm on the bleeding volume and infection rate, as well as medium- to long-term surgical outcomes.

Despite the aforementioned limitations, our findings showed a statistical power of more than 80% with a similar number of participants compared to similar studies, e.g., Brin et al. [6] and Gülenç et al. [9]. This result showed that the radiation exposure time could be reduced due to the reduced operation time by double C-arm use. However, there is a limitation in supporting our argument with the results derived from a small number of participants, a limitation mentioned in similar studies [6,9]. Our study can serve as a preliminary study for further study in confirming the positive effects of using a double C-arm in a large number of participants.

## 5. Conclusions

Despite the results obtained through a small number of participants, our study showed that CRIF surgery for FNF with double C-arm can reduce the time of radiation exposure by reducing the operation time compared to a single C-arm. This technique can also be used for other orthopedic surgeries to reduce radiation exposure time to minimize radiation exposure and protect patients and medical staff. However, further studies are needed to measure the radiation exposure time and dose of ionized radiation and to evaluate its potential impact on bleeding and infection rates.

## Figures and Tables

**Table 1:** Characteristics of the participants (*n* = 30).

	Single C-Arm Group (*n* = 14)	Double C-Arm Group (*n* = 16)	*p*-Value ^3^
Sex, man/woman, *n* (%)	10 (71.43)/4 (28.57)	8 (50)/8 (50)	0.232 ^2^
Age (years), mean (±SD)	48.57 ± 20.01	50.94 ± 6.21	0.677 ^1^
BMI (kg/m^2^) ^4^, mean (±SD)	22.22 ± 2.27	23.57 ± 3.07	0.189 ^1^
Anesthesia, general/spinal, *n* (%)	8 (57.15)/6 (42.85)	9 (56.25)/7 (43.75)	0.961 ^2^
Garden classification, stage 3/4, *n* (%)	5 (35.71)/9 (64.29)	11 (68.75)/5 (31.25)	0.070 ^2^

^1^ Independent sample *t*-test, ^2^ Chi-square test, ^3^ *p* < 0.05, ^4^ body mass index.

**Table 2:** Operation and radiation exposure times for both groups (single and double C-arm groups).

		Single C-Arm (*n* = 14)	Double C-Arm (*n* = 16)	*p*-Value ^3^
Radiation time (s)	Preparative	19.93 ± 9.02	14.25 ± 7.31	0.038 ^2^
	Intraoperative	80.50 ± 17.65	68.00 ± 14.23	0.034 ^2^
	Total	100.43 ± 24.59	83.06 ± 19.53	0.015 ^2^
Operation time (min)	Preparative	37.86 ± 6.99	28.75 ± 11.47	0.013 ^2^
	Intraoperative	55.36 ± 17.26	37.94 ± 6.55	0.002 ^2^
	total	93.21 ± 20.70	66.69 ± 13.97	0.001 ^1^

^1^ Independent sample *t*-test, ^2^ Mann–Whitney U test, ^3^ *p* < 0.05.

## Data Availability

No new data were created or analyzed in this study. Data sharing is not applicable to this article.

## References

[1] Sterling R.S. (2011). Gender and race/ethnicity differences in hip fracture incidence, morbidity, mortality, and function. Clin. Orthop. Relat. Res..

[2] Zuckerman J.D. (1996). Hip fracture. N. Engl. J. Med..

[3] Menzies I.B., Mendelson D.A., Kates S.L., Friedman S.M. (2012). The impact of comorbidity on perioperative outcomes of hip fractures in a geriatric fracture model. Geriatr. Orthop. Surg. Rehabilt..

[4] Boszczyk B.M., Bierschneider M., Panzer S., Panzer W., Harstall R., Schmid K., Jaksche H. (2006). Fluoroscopic radiation exposure of the kyphoplasty patient. Eur. Spine J..

[5] Mahajan A., Samuel S., Saran A.K., Mahajan M.K., Mam M.K. (2015). Occupational radiation exposure from C arm fluoroscopy during common orthopaedic surgical procedures and its prevention. J. Clin. Diagn. Res..

[6] Brin Y.S., Palmanovich E., Aliev E., Laver L., Yaacobi E., Nyska M., Kish B.J. (2014). Closed reduction and internal fixation for intertrochanteric femoral fractures is safer and more efficient using two fluoroscopes simultaneously. Injury.

[7] Giannoudis P.V., McGuigan J., Shaw D.L. (1998). Ionizing radiation during internal fixation of extracapsular neck of femur fractures. Injury.

[8] Kotil K., Sengoz A., Savas Y. (2011). Cervical transpedicular fixation aided by biplanar flouroscopy. J. Orthop. Surg..

[9] Gülenç B., Günaydin B., Çamur S., Talmaç M.A., Güler Y., Kara A. (2019). An effective technique in treatment of femoral neck fractures—Ostheosynthesis under double fluoroscopic guidance. Acta Chir. Orthop. Traumatol. Cechoslov..

[10] Bout C.A., Cannegieter D.M., Juttmann J.W. (1997). Percutaneous cannulated screw fixation of femoral neck fractures: The three point principle. Injury.

[11] Duffin M., Pilson H.T. (2019). Technologies for young femoral neck fracture fixation. J. Orthop. Trauma.

[12] Zhou X.Q., Li Z.Q., Xu R.J., She Y.S., Zhang X.X., Chen G.X., Yu X. (2021). Comparison of early clinical results for femoral neck system and cannulated screws in the treatment of unstable femoral neck fractures. Orthop. Surg..

[13] Huang S., Zhang Y., Zhang X., Zhou C., Li W., Wang Y., Zhu Z. (2023). Comparison of femoral neck system and three cannulated cancellous screws in the treatment of vertical femoral neck fractures: Clinical observation and finite element analysis. Biomed. Eng. Online..

[14] Schueler B.A., Vrieze T.J., Bjarnason H., Stanson A.W. (2006). An investigation of operator exposure in interventional radiology. Radiographics.

[15] Singer G. (2005). Occupational radiation exposure to the surgeon. J. Am. Acad. Orthop. Surg..

[16] Sanders R., Koval K.J., DiPasquale T., Schmelling G., Stenzler S., Ross E. (1993). Exposure of the orthopaedic surgeon to radiation. J. Bone Jt. Surg. Am..

[17] Beebe M.J., Jenkins P., Rothberg D.L., Kubiak E.N., Higgins T.F. (2016). Prospective assessment of the oncogenic risk to patients from fluoroscopy during trauma surgery. J. Orthop. Trauma.

[18] Mastrangelo G., Fedeli U., Fadda E., Giovanazzi A., Scoizzato L., Saia B. (2005). Increased cancer risk among surgeons in an orthopaedic hospital. Occup. Med..

[19] O’Rourke P.J., Crerand S., Harrington P., Casey M., Quinlan W. (1996). Risks of radiation exposure to orthopaedic surgeons. J. R. Coll. Surg. Edinb..

[20] Crawley M.T., Rogers A.T. (2000). Dose-area product measurements in a range of common orthopaedic procedures and their possible use in establishing local diagnostic reference levels. Br. J. Radiol..

[21] Peng K.T., Huang K.C., Chen M.C., Li Y.Y., Hsu R.W. (2006). Percutaneous placement of iliosacral screws for unstable pelvic ring injuries: Comparison between one and two C-arm fluoroscopic techniques. J. Trauma.

[22] Li Y.Y., Huang T.J., Cheng C.C., Hsu R.W. (2008). A comparison between one- and two-fluoroscopic techniques in percutaneous vertebroplasty. BMC Musculoskelet. Disord..

[23] Cho Y., Shin J.U., Kim S. (2023). Comparative study for osteosynthesis of femoral neck fractures: Cannulated screws versus femoral neck system. Hip Pelvis.

[24] Bailey J., Solan M., Moore E. (2022). Cone-beam computed tomography in orthopaedics. Orthop. Trauma.

[25] Sebaaly A., Jouffroy P., Moreau P.E., Rodaix C., Riouallon G. (2018). Intraoperative cone beam tomography and navigation for displaced acetabular fractures: A comparative study. J. Orthop. Trauma.

[26] Costa F., Tosi G., Attuati L., Cardia A., Ortolina A., Grimaldi M., Fornari M. (2016). Radiation exposure in spine surgery using an image-guided system based on intraoperative cone-beam computed tomography: Analysis of 107 consecutive cases. J. Neurosurg. Spine.

